# Millennial and orbital scale variability of the South American Monsoon during the penultimate glacial period

**DOI:** 10.1038/s41598-018-37854-3

**Published:** 2019-02-04

**Authors:** Stephen J. Burns, Lisa Kanner Welsh, Nick Scroxton, Hai Cheng, R. Lawrence Edwards

**Affiliations:** 1 Department of Geosciences, University of Massachusetts, Amherst, Massachusetts, 01003 USA; 2 Present Address: Geosyntec Consultants, 1111 Broadway, Oakland, CA 94607 USA; 3 Institute of Global Environmental Change, Xi’an Jiaotong University, Xi’an, 710049 China; 4 Department of Geology and Geophysics, University of Minnesota, Minneapolis, MN 55455 USA

## Abstract

The presence of large, rapid climate oscillations is the most prominent feature of the Earth’s last glacial period. These oscillations are observed throughout the Northern Hemisphere and into the Southern Hemisphere tropics. Whether similar oscillations are typical of prior glacial periods, however, has not been well established. Here, we present results of a study of the South American Summer Monsoon system that covers nearly the entire penultimate glacial period, from 195 to 135 ky BP. We use a well-dated, high-resolution (~50 y) time series of oxygen isotopes to show that the precession of the earth’s orbit is the primary control on monsoon intensity. After removing the precession signal we observe millennial oscillations that are very similar in amplitude and structure to the Dansgaard/Oeschger cycles of the last interglacial and that match well a synthetic reconstruction of millennial variability. Time series analyses shows that the most prominent of the observed cycles occur at considerably longer frequency (~3500 y) that the Dansgaard/Oeschger cycles from Marine Isotope Stages 2–4.

## Introduction

The last glacial period (about 115 to 15 ky BP) is marked by large, abrupt millennial-scale changes in climate that manifest themselves as temperature variability in the high northern latitudes and precipitation variability in the tropics. Termed Dansgaard/Oeschger (D/O) cycles, these events were first identified in the Greenland ice cores^1^ and have since been recognized in numerous climate records from both hemispheres^2–4^. Yet, because the Greenland ice cores do not extend beyond the previous interglacial period the nature of millennial scale climate variability during previous glacial periods remains highly uncertain. A number of marine^5–8^ and terrestrial records^9–11^ have noted the presence of millennial variability during Marine Isotope Stage 6 (135–190 ky BP), but they lack the resolution and/or chronological control for accurate intercomparison or to serve a template for other records. Speleothem records have been particularly valuable in characterizing millennial variability, but speleothem records that cover MIS 6 are rare and those that exist contain little information at the millennial scale. MIS 6 is largely absent from long speleothem records from the Amazon Basin^12^ and Chinese speleothem records^3,9,13^ are dominated by step-wise response to precessional variability, making extracting millennial variation difficult. Thus, no marine or terrestrial records have been produced with the requisite resolution and chronological control to produce a clear understanding of the number, timing or character of millennial-scale climate events beyond the last glacial period. Here we present a high resolution (~50 y), well-dated record of the orbital and millennial scale variability of the South American Summer Monsoon, derived from speleothems from the Peruvian Andes, that spans much of the penultimate glacial period, from 135 to 195 ky BP.

## Results and Discussion

Three stalagmites, P09-H1b, P10-H2 and P10-H5, were recovered from Huagapo Cave (11.27°S; 75.79°W) ~3,850 meters above sea level (masl) in the central Peruvian Andes. The samples were taken from three separate locations in the cave, all approximately 1000 to 1200 meters from the main entrance. The samples were cut into halves along the growth axis and polished. For Uranium series radiometric dating, 10, 11 and 16 subsamples, respectively, were taken parallel to growth layers (Table 1 Supplementary Material). For all samples, age dating errors are less than 0.5%. The samples grew from 135,650 y BP to 169,500 y BP (P09-H1b), 146,500 y BP to 177,400 y BP (P10-H5) and 169,550 to 196,600 y BP (P10-H2). All ages are in stratigraphic order and linear interpolation was used to establish age models for each sample (Fig. S1). For stable oxygen and carbon isotope analysis, 1,707 subsamples were taken at 0.2, 0.5 and 2 millimeter intervals, respectively, along the growth axis yielding an average time resolution of ~50 years for P09-H1b and H10-H2, and ~70 years for H10-H5. Oxygen isotope ratios are reported in per mille relative to the VPDB standard. Total analytical error, based on repeated analyses of standard materials, is 0.08 per mille (‰).

**Figure 1:**
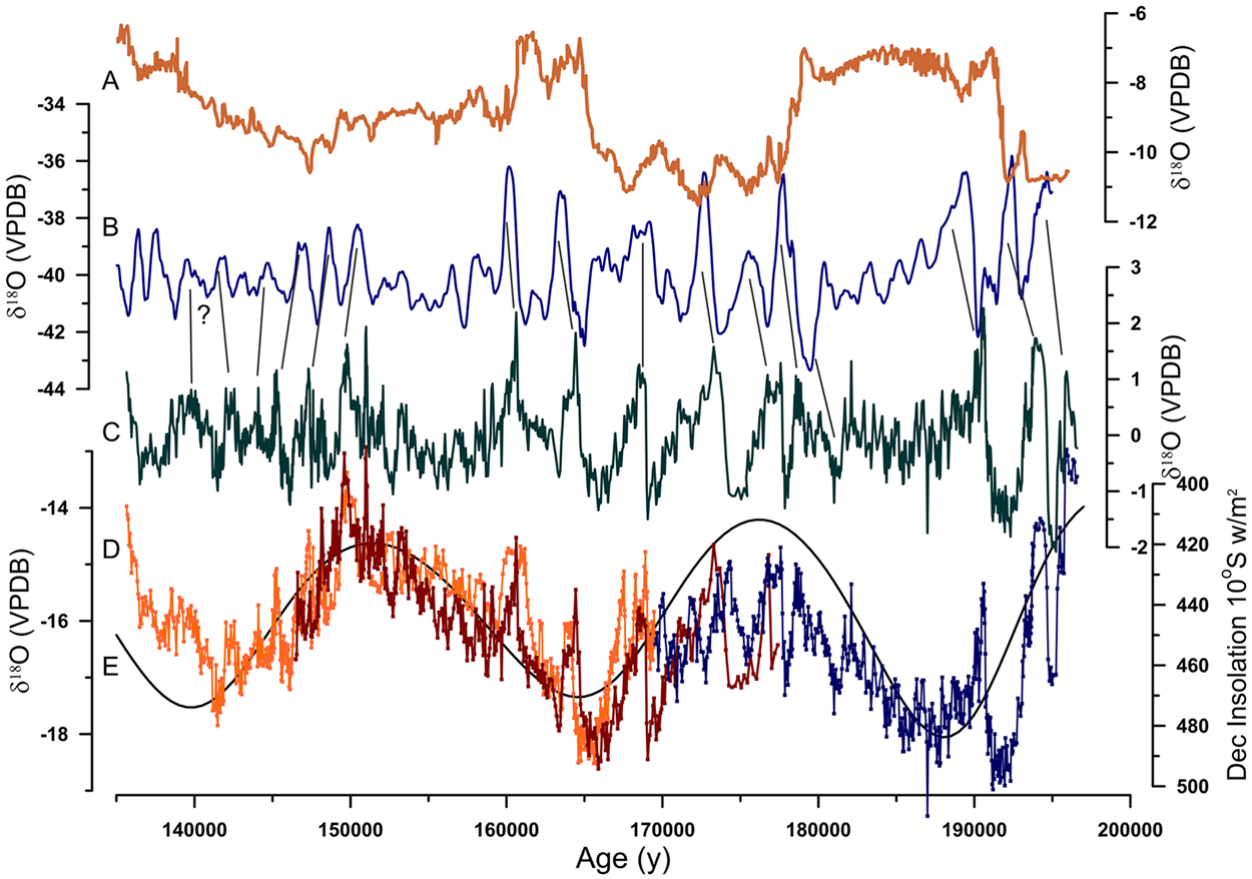
The stalagmite δ^18^O record of the East Asian monsoon from Sanbao Cave in China^3^ (**A**) and a synthetic reconstruction of millennial variability over the same time period^24^ (**B**) compared with the composite, detrended (**C**) and original and time series of δ^18^O from three stalagmites from Huagapo Cave in Peru (**D**). December insolation at 10°S^35^ (**E**) is also shown. For the detrended Huagapo record (**C**) a composite of the three samples was constructed and a 9th order polynomial fit was subtracted from it to remove orbital scale variability (and see Fig. S2).

Figure 1 shows the oxygen isotope time series for the samples. The δ^18^O values vary by over 6‰, ranging from −13.15‰ to −19.45‰. Sample H10-H5 overlaps the other two samples for almost the entire period of its growth and there is very good agreement in the isotopic time series from one sample to another except for the period from about 170 ky BP to 180 ky BP where slight age differences in similar patterns of variation appear. These differences, however, are within the errors of the age models. The time series begins at about 195 ky BP (equivalent to the end of MIS 7.1) and continues through MIS 6, the penultimate glacial period, and ending at 135 ky BP, just before the transition to interglacial period MIS5e. Variations in δ^18^O of speleothem calcite record variations in δ^18^O of cave drip waters (precipitation) and cave temperature, and may additionally be affected by kinetic isotope fractionation during CO_2_ degassing or evaporation of water during calcite deposition^14^. The excellent replication of δ^18^O in our time series by different stalagmites is a strong indication that kinetic isotope effects and evaporation of drip waters are of minor importance. Changes in cave temperature are surely incorporated into our δ^18^O record through the equilibrium fractionation between water and calcite^15^. In our record, however, temperature variation would serve to dampen the amplitude of isotopic variability because warm climatic periods, such as equivalent to MIS 7.1, occur when the isotopic time series has the least negative values. Changes in the isotopic composition of seawater could also be incorporated into our record. The δ^18^O value of seawater is generally higher during colder periods when more ^18^O-depleted ice is stored on the continents. Yet, colder periods are when the δ^18^O values of our speleothems is low, indicating that changes in seawater isotopic composition would, as for temperature, serve to decrease the amplitude of isotopic variability in our record. Thus, we interpret the primary climate signal recorded in our samples to be changes in the δ^18^O of precipitation at the cave site.

Variations in the isotopic composition of rainfall in the central Andes are related to climate as follows. The moisture source for precipitation in the central Peruvian Andes is the tropical Atlantic Ocean. Prevailing easterly trade winds during the austral summer carry moisture to and across the Amazon Basin and ultimately into Chaco Low where they are uplifted to the Andes^16^. Today, about 65% of annual rainfall in the central Andes occurs during the austral summer, December-March (DJFM), rainy season. During the SASM, moisture from the tropical Atlantic is transported across the continent before being uplifted to the Andes mountains^16^. Rainout of the heavy isotope during moisture transport results in isotopically depleted rainfall in the Amazon Basin due to an ‘amount effect’. A more vigorous SASM results in greater rainout of the heavy isotope and more negative δ^18^O values in tropical South American rainfall^17^. Because the Amazon Basin^16^ is source or moisture for the Peruvian Andes, this relationship is also observed in rainfall in the Andes. Thus, precipitation δ^18^O at the study site is strongly anti-correlated to rainfall amount in the Amazon Basin^18,19^.

On orbital timescales an additional factor that might affect precipitation δ^18^O is changing seasonality of precipitation. An increase in the portion of winter precipitation could lead to an increase in the δ^18^O of precipitation independent of changes in SASM. In a modeling study Liu and Battisti^20^ found that in the northern Andes changes in the seasonality of precipitation contribute significantly to the total change in precipitation δ^18^O. Their study also found that degree of rainout in the SASM strongly impacts the isotopic composition of precipitation delivered downstream over the Andes in agreement with prior work. It is difficult to quantitatively assess the possible contribution of changes in seasonality to our record, but models of both modern climatological controls on the δ^18^O of precipitation and those on orbital timescales all indicate that our records are strongly impacted by and record SASM intensity integrated along the path of moisture transport from the eastern Amazon Basin to the Andes^17–20^.

The first order variations in δ^18^O values of the record closely parallel summer solar insolation in the Southern Hemisphere, which is dominated by precession in earth’s orbit (Fig. 1). Low δ^18^O values, indicative of a stronger SASM and greater precipitation are associated with greater insolation. Increased summer insolation results in increased sensible heating of the land surface, an increased land-sea thermal contrast and intensified monsoonal circulation. Our results are in good agreement with previous studies of the SASM on orbital timescales^12,21^. Our record is antiphased with records of Northern Hemisphere monsoon records at orbital timescales, which indicates an additional factor leading to increases and decreases in SASM intensity: changes in the mean latitudinal position of the Atlantic Intertropical Convergence Zone (ITCZ). A more southerly mean position of the ITCZ results in greater precipitation during the SASM^21^. A notable difference exists, however, in the recorded response of Northern Hemisphere versus Southern Hemisphere monsoons to insolation changes. The existing long Northern Hemisphere monsoon records, from the Asian and East Asian Monsoon regions, show a highly nonlinear response to precession driven changes in insolation, with a rectangular step function shape (Fig. 1a) to the records^3,9,13^. In contrast, the SASM shows a nearly linear response to insolation forcing during the last interglacial^21,22^ and, as shown here, during the previous interglacial. A linear response of the monsoons to orbital forcing is predicted by general circulation models and the non-linear response of the EASM has been interpreted as indicating that some additional forcing, such as sea surface temperature or changes in moisture source, must be incorporated into the EASM records^23,24^.

The δ^18^O time series also shows considerable variability at millennial timescales. To better study these variations we removed the orbital-scale variability by subtracting a 9th order polynomial fit to the data from the original time series. The resultant detrended time series (Fig. 1) shows a pattern of variations that is very similar in amplitude and style to D/O cycles recognized in Greenland ice cores and many other records from MIS 2–4. In particular, for the period from 160 ky BP to 180 ky BP these cycles show gradually increasing strength of the SASM ended by a sudden decrease in SASM strength, a sawtooth pattern that is suggestive of the D/O cycles of MIS 3 in the Greenland ice cores. In previous work in nearby Pacupahuain Cave, 5 km from Huagapo, δ^18^O values of a stalagmite show a pattern of millennial events closely matching those from Greenland ice cores^4^ demonstrating that the isotopic values of rainfall in the study area responded strongly to high northern latitude forcing during the Last Interglacial. Cold, stadial, periods recorded in the Greenland ice cores coincide with enhanced precipitation in South America, while Greenland interstadials are dry^4^. The amplitude of the MIS3 events in Peru, 2 to 2.5‰, is also similar to the amplitude of the MIS6 events found in this study. Thus, we suggest that our detrended isotopic time series is an accurate record of the timing and nature of millennial variability and high northern latitude forcing of climate during MIS 6.

To confirm this relationship, we searched for other well-dated, high-resolution paleoclimate data sets that with which to compare our data. While the best of these, from Chinese speleothems^2,3^ and marine records from the Iberian margin^5^, show some similar millennial variability, none are of sufficient resolution with strong enough chronological control to make a definitive direct comparison. This lack of high-resolution records of millennial variability from previous glacial periods led Barker *et al*.^25^ to construct a synthetic record using Antarctic ice core data. A comparison of our detrended record with the synthetic record of Barker *et al*. shows a remarkable similarity in the overall pattern (Fig. 1). The timing of individual millennial events varies by up to 1500 y (though generally less) possibly because of the relatively large errors in absolute age control on the Antarctic ice cores on which the Barker *et al*. reconstruction is based. Yet, the number of high amplitude D/O style cycles and the relative timing and spacing of events is much the same.

**Figure 2:**
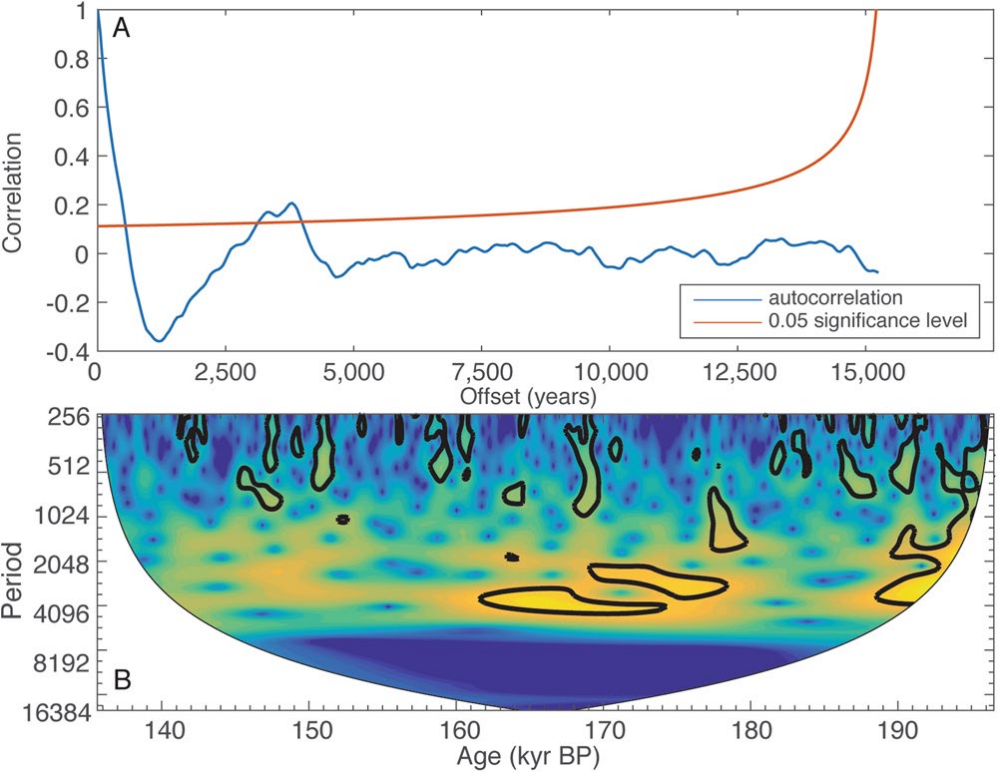
Top panel shows the autocorrelation of the detrended composite record (blue) and the 95% confidence interval (red) (p = 0.05 significance level). Significant peaks are located at 3350 and 3800 ky. Lower panel is a continuous wavelet transform, using a morlet wave. Thick black line encompasses areas of >95% significance.

One fundamental difference in the nature of millennial events in MIS 3 from MIS 6 is the periodicity. Numerous studies have suggested that D/O events of the last glacial period occur approximately every 1500 years^26,27^. This frequency is particularly strongly expressed between 13 and 46 kyBP^28^. Bond *et al*.^29^ found a similar periodicity in ice-rafted debris deposits over the Holocene and concluded that there is a persistent solar influence on climate in the North Atlantic region. While the periodic nature of D/O events has been questioned^30^, as has any relationship to solar forcing^31^, the concept of a quasi-periodic D/O cycling of around 1500 years persists. We find that the millennial-scale events from MIS 6 are also occur at periodic intervals for a part of our record, but those intervals more that twice as long as observed during MIS3. Spectral analyses of the detrended isotopic time series from MIS 6 (Fig. 2) suggest a dominant periodicity of around 3500 years. Wavelet analysis indicates that this period is significant only between 160 ky BP and 180 ky BP, during which the five largest millennial-scale events in the record are found. We know of no recognized climate forcing at or near this frequency. The lack of any clear external forcing mechanism and the large difference between the time intervals between D/O events in MIS 3 and MIS 6 lead us to suggest that a mechanism tied to ice sheet dynamics, not any external forcing, results in an apparent quasi-periodicity in these records. The differences between MIS 3 and 6 could than be tied to differences in nature of the ice sheets in different glacial periods.

## Methods

Speleothems P09-H1b, P10-H2 and P10-H5 were collected from Huagapo Cave in the central Peruvian Andes. The study site has mean annual rainfall of 690 mm, with about 65% falling during the austral summer (Peruvian Meteorological Service, SENAMHI, unpublished data, 1919–1999). Seasonal temperature variation is small, average summer temperatures in the central Peruvian Andes at 3750 m are 11 °C compared to winter temperatures of 9 °C at the same elevation (SENAMHI). Cave air temperatures at the time of sampling were 12 °C, relative humidity was not measured. Huagapo Cave is 2,844 meters in length and was formed within a Triassic dolomitic limestone massif^32^. Stalagmites P09-H1b, P10-H2 and P10-H5 are 13.5, 22 and 109 cm tall, respectively. All samples are 100% calcite as confirmed by X-ray diffraction analysis. Each stalagmite was cut into halves along the growth axis and polished. Subsamples of approximately 100 milligrams were milled parallel to growth layers for radiometric dating. For stable oxygen and carbon isotope analysis, subsamples of approximately 200 micrograms were taken as along the center of the growth with a 0.6 mm drill bit.

The radiometric dates (Supplementary Table 1) were measured using a multi-collector, inductively coupled plasma mass spectrometry (MC-ICPMS) on a Thermo-Finnigan Neptune at the Minnesota Isotope Laboratory with procedures similar to those described in^33^. The stable isotopic analyses were performed at the University of Massachusetts using an on-line carbonate preparation system linked to a Finnigan Delta Plus XL ratio mass spectrometer. Results are reported as the per mil difference between sample and the Vienna Pee Dee Belemnite standard in delta notation where δ^18^O = (R_sample_/R_standard_ − 1) ∗ 1000, and R is the ratio of the minor to the major isotope. Reproducibility of the standard materials is better than 0.08‰. Values are reported relative to the VPDB standard.

A time-series analysis of the oxygen isotope record was conducted on an equally spaced fifty-year composite record filtered to remove orbital scale variability. The composite was created by taking an annual interpolation using a piecewise cubic hermite interpolating polynomial, then averaging to fifty-year bins. A 5^th^ order Butterworth high pass filter with a frequency of 5000 years was then applied to remove the first order orbital scale signal.

Two time-series analyses were then conducted on the filtered composite record. First, an autocorrelation, with significance-level calculations including red-noise of the composite record was done. Butterworth and autocorrelation calculations were conducting using MATLAB functions created by Dave Heslop, now at The Australian National University. Second, a continuous wavelet transform using a Morelet wavelet to determine spectral relationships in the time-frequency space^34^.

All data will be made available online through the NOAA National Centers for Environmental Information (https://www.ncdc.noaa.gov/data-access/paleoclimatology-data/datasets).

## Supplementary information

Supplementary material
