# Supplementary material for: Millennial and orbital scale variability of the South American Monsoon during the penultimate glacial period
Source: Sci Rep. 2019 Feb 4;9:1234. doi: 10.1038/s41598-018-37854-3 (PMC6362059; doi:10.1038/s41598-018-37854-3)
Supplement: Supplementary file 1 — Supplementary material [file 41598_2018_37854_MOESM1_ESM.pdf]

Supplementary material, Burns, S.J., Kanner Welsh, L., Scroxton, N., Cheng, H. and Edwards, R.L., "Millennial and orbital scale variability of the South American Monsoon during the penultimate glacial period"

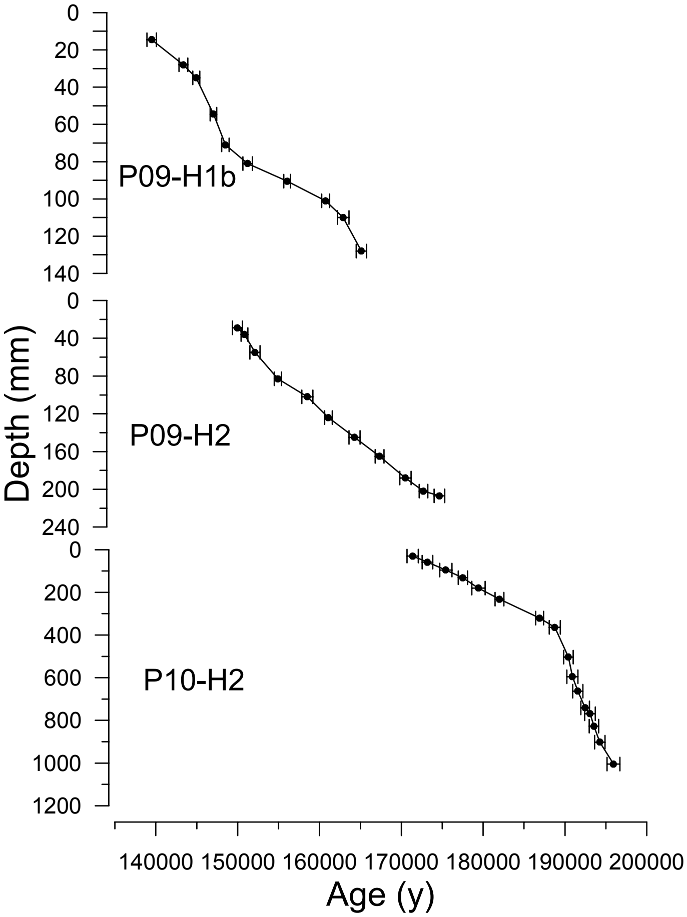

Supplementary Figure 1.
Age versus depth for samples P09-H1b, P09-H2 and P10-H2. Error bars for Th230/U ages are shown (data in Supplementary Table T1). Linear interpolation between age determinations was used to construct age models.

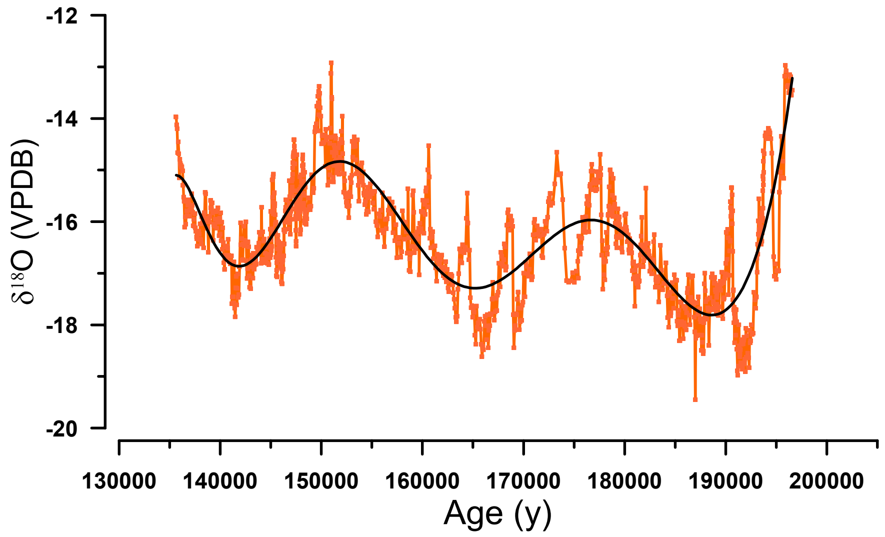

Supplementary Figure 2.
Composite δ18O time series with 9th order polynomial fit to the composite data set. The polynomial fit was subtracted from the composite data to produce the detrended data set shown in Figure 1.

Polynomial Equation Y = -209982399 + 11385.3 * X - 0.273580 * (X^2) + 3.82391E-006 * (X^3) - 3.426304E-011 * (X^4) + 2.04102E-016 * (X^5) - 8.08332E-022 * (X^6) + 2.05247E-027 * (X^7) - 3.03205E-033 * (X^8) + 1.98559E-039 * (X^9)

Table S1 ^{230}Th dating results. The error is 2s error.

Sample Depth (mm)	^{238}U (ppb)	^{232}Th (ppt)	^{230}Th / ^{232}Th (atomic x10^{-6})	δ^{234}U* (measured)	^{230}Th / ^{238}U (activity)	^{230}Th Age (yr) (uncorrected)	^{230}Th Age (yr) (corrected)	$\delta^{234}U_{Initial}$** (corrected)	^{230}Th Age (yr BP)*** (corrected)
P09H1b -									
14.5	885.2 ±1.5	11594 ±233	5402 ±109	4203.1 ±4.7	4.2911 ±0.0099	139611 ±580	**139499 ±581**	63125 ±10	**139439 ±581**
28	633.5 ±0.7	5825 ±117	7837 ±157	4206 ±3	4.3707 ±0.0063	143831 ±389	**143794 ±533**	6312 ±9	**143732 ±533**
35	501 ±1	6404 ±128	5644 ±113	4190.1 ±4.7	4.3783 ±0.0061	145057 ±414	**145006 ±416**	6309 ±10	**144946 ±416**
54.5	373.4 ±0.4	2413 ±48	11227 ±225	4174.2 ±4.5	4.4010 ±0.0057	147139 ±398	**147113 ±399**	6322 ±10	**147053 ±399**
71	466 ±1	1374 ±28	24662 ±495	4155.8 ±4.7	4.4098 ±0.0068	148582 ±462	**148570 ±462**	6320 ±11	**148510 ±462**
81	356.3 ±0.4	7534 ±151	3410 ±68	4061 ±3	4.3728 ±0.0063	151380 ±423	**151295 ±578**	6224 ±9	**151233 ±578**
90.5	570.7 ±0.6	2126 ±43	19148 ±384	3924.0 ±3.6	4.3256 ±0.0054	156125 ±404	**156110 ±404**	6096 ±9	**156050 ±404**
101	518.3 ±0.6	4563 ±91	8129 ±163	3862.3 ±4.2	4.3409 ±0.0061	160846 ±485	**160810 ±485**	6080 ±11	**160750 ±485**
110	737.4 ±0.8	1581 ±32	33175 ±667	3801 ±3	4.3152 ±0.0072	162960 ±537	**162951 ±700**	6021 ±10	**162889 ±700**
128	855.6 ±1.2	3101 ±62	19745 ±397	3794.5 ±3.8	4.3409 ±0.0083	165198 ±635	**165124 ±635**	6031 ±11	**165064 ±635**
P09H2 -									
28.5	292 ±0	718 ±14	29253.8 ±588.9	4076.2 ±4.4	4.3642 ±0.0066	150047 ±460	**150037 ±610**	6225 ±11	**149975 ±610**
36	365.9 ±0.4	2128 ±43	12314 ±247	4053.6 ±4.4	4.3750 ±0.0055	150913 ±409	**150890 ±409**	6223 ±10	**150830 ±409**
55	369 ±0	193 ±4	136837.7 ±2847.6	4014.9 ±4.2	4.3450 ±0.0066	152190 ±463	**152187 ±616**	6169 ±10	**152125 ±616**
83	483.0 ±0.6	818 ±16	42195 ±849	3952.5 ±4.1	4.3338 ±0.0060	154994 ±445	**154986 ±445**	6121 ±10	**154926 ±445**
102	422 ±0	265 ±5	112877.4 ±2310.8	3851.9 ±3.9	4.2975 ±0.0067	158584 ±505	**158581 ±664**	6026 ±11	**158519 ±664**
124	422.1 ±0.5	615 ±12	48091 ±969	3761.8 ±3.9	4.2525 ±0.0059	161164 ±477	**161157 ±477**	5928 ±10	**161097 ±477**
145	687 ±1	743 ±15	64466.0 ±1297.9	3685.4 ±3.5	4.2268 ±0.0065	164352 ±521	**164347 ±686**	5860 ±10	**164285 ±686**
165	582.7 ±0.8	364 ±7	109616 ±2223	3572.6 ±3.7	4.1757 ±0.0062	167419 ±542	**167416 ±542**	5746 ±11	**167356 ±542**
188	451.1 ±0.5	228 ±5	135872 ±2819	3528.7 ±3.6	4.1618 ±0.0058	170557 ±519	**170554 ±690**	5710 ±10	**170492 ±690**
202	504.4 ±0.6	3475 ±70	9846 ±197	3449.0 ±3.6	4.1137 ±0.0055	172795 ±521	**172764 ±521**	5616 ±10	**172704 ±521**
207	733.4 ±1.1	22834 ±458	2162 ±43	3393.0 ±4.1	4.0826 ±0.0068	174659 ±646	**174520 ±653**	5552 ±12	**174460 ±653**
P10-H2 -									
30	484.6 ±0.6	1155 ±23	30315 ±609	3745 ±3	4.3811 ±0.0063	171462 ±526	**171452 ±698**	6076 ±11	**171390 ±698**
58.5	568.1 ±0.6	398 ±8	91110 ±1859	3206.2 ±3.5	3.9088 ±0.0066	173271 ±648	**173267 ±648**	5258 ±11	**173207 ±648**
95	555.5 ±0.7	42 ±1	860109 ±27849	3219 ±3	3.9237 ±0.0057	175498 ±570	**175497 ±746**	5282 ±10	**175435 ±746**
135	628 ±1	748 ±15	53377 ±1075	3143.3 ±4.2	3.8848 ±0.0048	177585 ±567	**177579 ±567**	5203 ±11	**177519 ±567**
180	582.6 ±0.8	114 ±3	332011 ±7446	3172 ±4	3.9117 ±0.0062	179492 ±640	**179491 ±819**	5249 ±11	**179429 ±819**
232	548 ±1	237 ±5	144464 ±2999	3010.1 ±3.7	3.7910 ±0.0041	182061 ±533	**182059 ±533**	5032 ±10	**181999 ±533**
321	640 ±1	267 ±6	146007 ±3021	2857.7 ±2.8	3.6883 ±0.0036	186969 ±475	**186966 ±475**	4843 ±8	**186906 ±475**
364	670 ±1	116 ±3	350275 ±7756	2843.0 ±3.6	3.6913 ±0.0053	188821 ±681	**188820 ±681**	4844 ±11	**188760 ±681**
503	844 ±1	207 ±4	246326 ±5291	2804.1 ±3.3	3.6675 ±0.0041	190481 ±575	**190479 ±575**	4800 ±10	**190419 ±575**
595	639 ±1	27 ±2	1357343 ±76299	2675.1 ±4.0	3.5411 ±0.0043	190969 ±667	**190969 ±667**	4586 ±11	**190909 ±667**
663	715 ±1	325 ±7	129327 ±2660	2693.1 ±3.6	3.5653 ±0.0041	191631 ±615	**191628 ±615**	4625 ±10	**191568 ±615**
741	622 ±1	1315 ±26	27906 ±560	2705.4 ±2.8	3.5814 ±0.0038	192541 ±531	**192529 ±531**	4651 ±8	**192469 ±531**
768.5	485 ±0	1190 ±24	24166 ±485	2711.8 ±3.1	3.5976 ±0.0048	193134 ±652	**193121 ±652**	4677 ±10	**193061 ±652**
827.5	676 ±1	195 ±4	204836 ±4339	2700.3 ±3.2	3.5901 ±0.0039	193602 ±577	**193600 ±577**	4663 ±9	**193540 ±577**
902	583 ±1	997 ±20	34469 ±692	2678.1 ±3.9	3.5693 ±0.0044	194331 ±674	**194322 ±674**	4628 ±11	**194262 ±674**
1005	1498.5 ±2.0	1553 ±31	57194 ±1148	2685.4 ±3.2	3.5956 ±0.0059	195997 ±784	**195991 ±783**	4669 ±12	**195931 ±783**

*$\delta^{234}U = ([^{234}U/^{238}U]_{activity} - 1)x1000$. ** $\delta^{234}U_{initial}$ was calculated based on ^{230}Th age (T), i.e., $\delta^{234}U_{initial} = \delta^{234}U_{measured}$ x e^{l234xT}.
Corrected ^{230}Th ages assume the initial $^{230}Th/^{232}Th$ atomic ratio of 4.4 ±2.2 x10^{-6}.   Those are the values for a material at secular equilibrium, with the bulk earth $^{232}Th/^{238}U$ value of 3.8. The errors are arbitrarily assumed to be 50%.
***B.P. stands for "Before Present" where the "Present" is defined as the year 1950 A.D.
